# Comparative Analysis of Artifact Expression in Zirconia and Graphene Crowns in CBCT Images From Different Systems

**DOI:** 10.1155/ijod/4412687

**Published:** 2026-03-11

**Authors:** Sâmia Mouzinho-Machado, Rocharles Cavalcante Fontenele, Fernanda Bulhões Fagundes, Bahaaeldeen M. Elgarba, Reinhilde Jacobs, Sergio Lins de-Azevedo-Vaz

**Affiliations:** ^1^ State University of Campinas, Campinas, Brazil, unicamp.br; ^2^ Department of Stomatology, Public Health and Forensic Dentistry, Division of Oral Radiology, School of Dentistry of Ribeirão Preto, University of São Paulo, Ribeirão Preto, São Paulo, Brazil, usp.br; ^3^ OMFS-IMPATH Research Group - Department of Imaging and Pathology, Faculty of Medicine, University Hospitals Leuven, Katholieke Universiteit Leuven, Leuven, Belgium, uzleuven.be; ^4^ Department of Prosthodontics, Faculty of Dentistry - Tanta University, Tanta, Egypt; ^5^ Department of Dental Medicine, Karolinska Institute, Stockholm, Sweden, ki.se; ^6^ Department of Clinical Dentistry, Federal University of Espírito Santo, Espírito Santo, Brazil, ufes.br

## Abstract

**Introduction:**

Comparing artifact expression in emerging materials, like zirconia, known for high artifact generation, to lower‐density materials like graphene is essential to find ways to minimize it. Evaluating these materials across cone‐beam computed tomography (CBCT) systems and acquisition protocols provides a comprehensive performance assessment.

**Purpose:**

To compare zirconia and graphene‐reinforced crowns’ volumetric alteration artifact, surface area distortion, and general artifact expression on CBCT images using three systems and three protocols.

**Materials and Methods:**

An anthropomorphic phantom covered with Mix‐D soft tissue‐simulator material was scanned using 3 CBCT systems: 3D Accuitomo 170, NewTom VGi evo, and Veraview X800. A single zirconia or graphene crown was placed on the right mandibular second premolar. Three acquisition protocols were used: medium field of view (FOV) with standard resolution (SR), small FOV with SR, and small FOV with high resolution (HR). The CBCT images of the crowns were segmented and analyzed using the 3‐matic Medical 17.0 (Materialise) software program to obtain the crown’s volume and surface area. The root mean square error (RMSE) between the segmentation results and a reference standard (micro‐CT scanning) was used to measure general artifact expression. A two‐way repeated measures analysis of variance (ANOVA) was performed to assess the parameters’ influence in the volumetric alteration artifact, surface area distortion, and general artifact expression.

**Results:**

The zirconia crown exhibited significantly more volumetric alteration artifact, surface area distortion, and general artifact expression than the graphene crown across all CBCT systems (*p* < 0.05). For the Veraview X800 system and both materials, the medium FOV protocol showed greater general artifact expression compared with the small FOV with SR protocol (*p* < 0.05).

**Conclusions:**

Zirconia’s greater volumetric alteration, surface detail distortion and loss, and artifact expression in CBCT imaging can be mitigated by using lower‐density materials like graphene. Furthermore, optimizing CBCT exposure protocols may independently reduce artifacts and surface distortion, regardless of the material’s density.

## 1. Introduction

Artifacts are distortions or errors present in reconstructed images that do not accurately represent the morphological characteristics of the scanned object [1, 2]. Among these, volumetric alteration artifacts are characterized by changes in the volume of high‐density materials, often referred to as blooming when the volume is increased [3, 4]. The volume changes are usually heterogeneous, which can lead to object shape variation and detail loss. In general, volumetric alteration artifacts are associated with beam hardening and partial volume effects [5], where the atomic number and physical density of materials influence artifact expression, with higher values typically resulting in more pronounced artifacts [6–8]. Furthermore, artifacts, in general, interfere with image quality [9–11] and diagnostic accuracy [12–14], while volumetric alteration artifacts may obscure adjacent structures, such as thin buccal peri‐implant bone thickness [4].

Previous studies on volumetric alterations have often focused on implants or solid cylindrical objects [3, 7, 15–17]. However, since volumetric alteration artifact can distort the shape of objects in a heterogeneous way, irregular materials commonly used in oral rehabilitation, such as dental crowns, may be affected in diverse ways. This highlights the importance of analyzing volumetric alterations across different types of dental objects.

The use of crowns in oral rehabilitation can achieve extended longevity under specific conditions [18, 19], with material selection critically influencing the treatment. Currently, various alloys are used clinically, including chromium–cobalt, silver–palladium, nickel–chromium, titanium, and zirconia alloys. However, some materials have drawbacks, for example, titanium has allergenic potential [20], and most alloys hinder the visualization of cone‐beam computed tomography (CBCT) images by introducing artifacts [21–23].

Zirconia is commonly used in prosthetic restorative treatments, offering numerous advantages for dentistry [24, 25]. However, zirconia exhibits high radiopacity in radiological exams [24, 26] due to its high density and atomic number [24]. Therefore, it is important to explore alternative materials, such as graphene, with reduced potential for artifact expression. Graphene is an extremely resistant carbon composite and low‐density material used for various purposes, from manufacturing electronic devices to medical rehabilitation components [27]. Previous studies have demonstrated its potential use in different dental applications due to its biocompatibility, increased strength, elasticity, and mechanical properties, and antibacterial activity [27, 28]—including its use as material for crown confection when mixed with a biopolymer [29–34]—as well as its relative radiolucency and hypodensity in imaging exams compared to other materials currently used in dentistry [27, 35]. A thermoplastic acrylic disc made with polymethylmethacrylate (PMMA) and reinforced with graphene, G‐CAM, is commercialized as a material for many restorative purposes, including the manufacture of implant overdentures and crowns [36]. The company responsible for its development highlights, among other characteristics, its biocompatibility, wide chromatic range, release of minimum residual monomer, high modulus and elastic limit, and high deformation resistance and stress limit [36]. In fact, the effects of surface treatments on the retentive strengths of crowns fabricated with this material have also been explored, further indicating the potential use of graphene in dentistry [37]. Although there is no information regarding this material’s radiolucency in imaging exams, PMMA is a polymer composed mainly of carbon, which has a low atomic number (*Z* = 6) [38] and, therefore, also tends to generate a low amount of artifacts.

As described, object composition [7, 16, 17] significantly contribute to artifact generation on CBCT images, as well as specific CBCT acquisition protocols [8, 16, 39, 40], while variations in the CBCT system [15] itself can further affect volumetric alteration artifact expression. Voxel and field of view (FOV) sizes are some of the acquisition protocols that can affect artifact expression, with evidence of voxel size increase leading to higher artifact expression [16]. For FOV size, there is conflicting evidence regarding the artifact generation in CBCT images acquired with smaller or larger FOVs [15, 16, 39, 40].

Therefore, the aim in this study was to compare the volumetric alteration artifact, surface area distortion, and general artifact expression produced by zirconia and graphene‐reinforced crowns as a pilot material for crown confection on three CBCT systems under three protocols. The hypotheses were: zirconia crowns would exhibit more volumetric alteration artifact, surface area distortion, and general artifact expression than graphene‐reinforced crowns, and larger FOVs and voxel sizes would result in increased volumetric alteration artifact, surface area distortion, and general artifact expression.

## 2. Materials and Methods

This ex vivo study was conducted in compliance with the Declaration of Helsinki, with ethical approval obtained from the Belgian National Council for Bioethics Research Committee (Protocol Number: NH019 2019‐09‐03).

A dry skull with dentate jaws covered with Mix‐D to simulate a patient’s soft tissues was prepared for use in the anthropomorphic phantom (Figure 1) [41]. A prosthetic dentistry (B.M.E) expert prepared the right mandibular second premolar for crown placement (Figure 2A). Subsequently, the expert scanned the mandible with prepared teeth using an intraoral scanner (Trios 3; 3Shape). The resulting three‐dimensional (3D) image was exported in stereolithography (STL) file format for import into the Exocad software program (DentalCaAD 3.1 Rijeka, Exocad GmbH) for virtual crown design. The crown design specifications included a minimum thickness of 0.4 mm at the margins and 0.6 mm on the occlusal surface.

Figure 1Phantom made with dry skull and dentate jaws covered with Mix‐D. (A) Side view. (B) Front view. (C) Posterior view.

**Figure:**
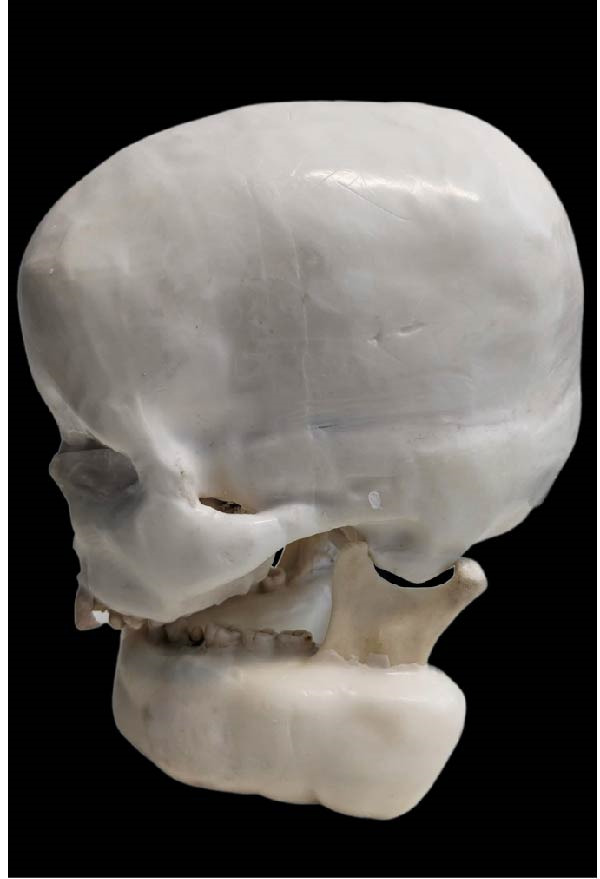
(A)

**Figure:**
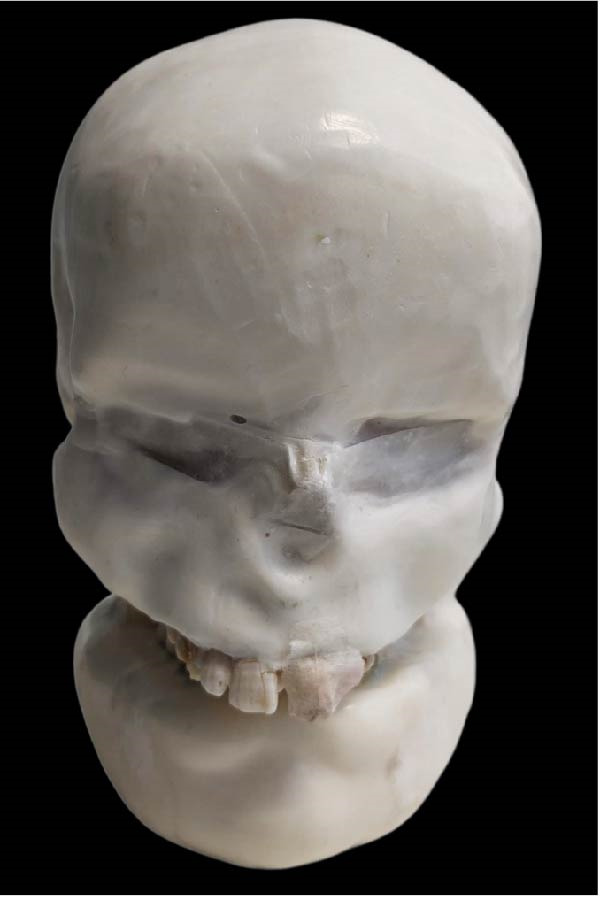
(B)

**Figure:**
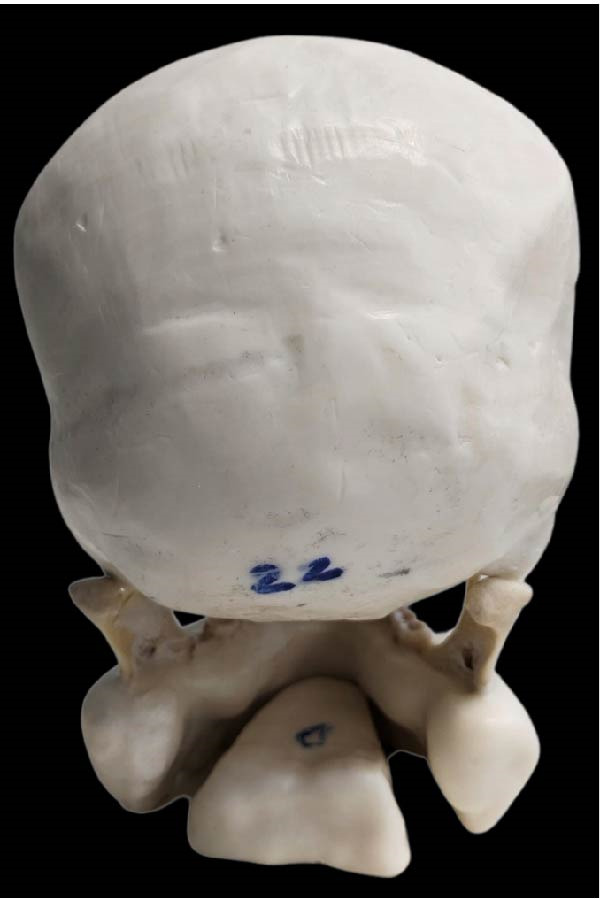
(C)

Figure 2Dry dentate mandible. (A) Right mandibular second premolar prepared for rehabilitation with prosthetic crown. (B) Graphene‐reinforced crown positioned on the right mandibular second premolar. (C) Zirconia crown positioned on the right mandibular second premolar.

**Figure:**
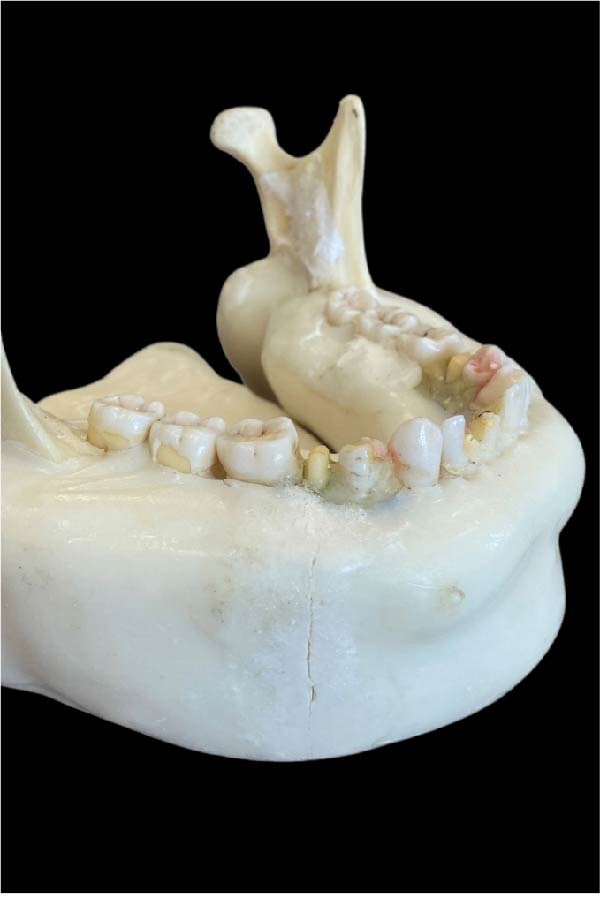
(A)

**Figure:**
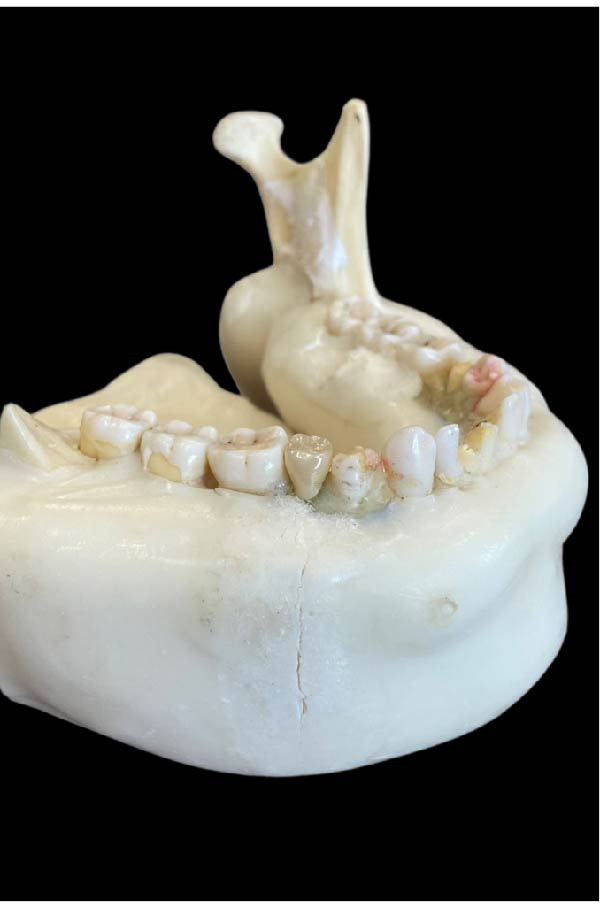
(B)

**Figure:**
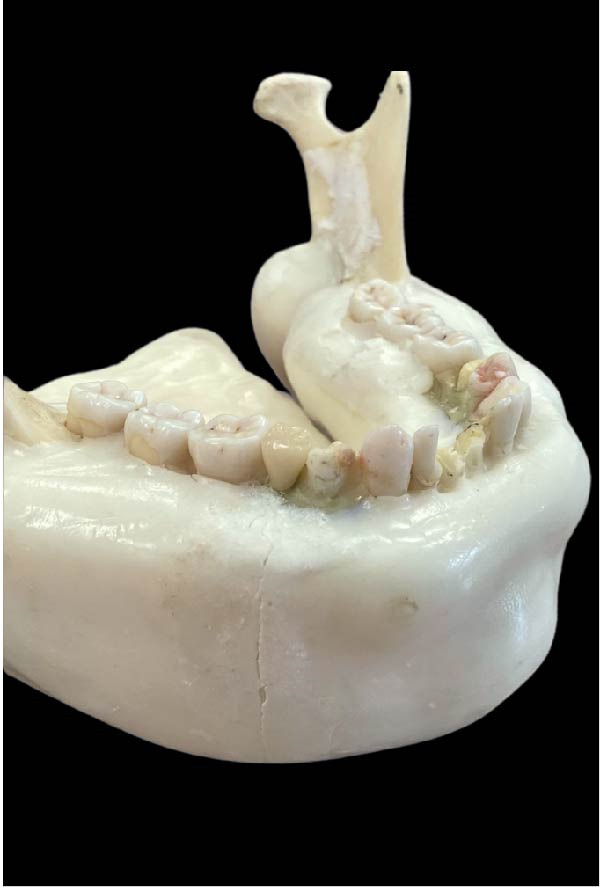
(C)

Two materials were used for crown production: graphene‐reinforced biopolymer (G‐CAM; Graphenano Dental) (Figure 2B) and zirconia (KATANATM Zirconia Blocks; Kuraray Noritake Dental Inc) (Figure 2C). The same design specifications were applied to both materials to guarantee uniform size and thickness of the crowns. All crowns were exported in STL format and sent to the laboratory for milling (Straumann M series milling machine, Amann Girrbach AG). Finally, a prosthodontist (B.M.E) verified the fit of each crown after milling to ensure proper positioning on the prepared tooth.

Image acquisition of the phantom included three different CBCT systems (3D Accuitomo 170 [J. Morita], NewTom VGi evo [Cefa Dental Group], and Veraview X800 [J. Morita]) due to their limited artifact expression [15] or adequate visualization of fine endodontic structures [42]. For each system, there were three acquisition protocols with parameters as closely similar as possible: medium FOV with standard resolution (SR), small FOV SR, and small FOV with high resolution (HR) (Table 1). For the former, the center of the FOV was positioned equidistantly between the mandibular second premolars, while for the small FOVs it was positioned in the middle of the scanned premolar. For all CBCT systems, the images were acquired without the use of a metal artifact reduction (MAR) algorithm.

**Table 1:** Acquisition protocols and parameters for each studied cone‐beam computed tomography system.

CBCT systems	Tube voltage (kVp)	Tube current (mA)	Exposition time (s)	FOV (cm)	Voxel size (mm)
Medium FOV–standard resolution					
3D Accuitomo 170	89	5	17.5	8 × 8	0.125
NewTom VGi evo	110	3	4.32	8 × 8	0.125
Veraview X800	90	5	17.86	8 × 8	0.125
Small FOV–standard resolution					
3D Accuitomo 170	89	5	17.5	4 × 4	0.125
NewTom VGi evo	110	3	4.32	5 × 5	0.125
Veraview X800	90	5	17.86	4 × 4	0.125
Small FOV–high resolution					
3D Accuitomo 170	89	5	30.8	4 × 4	0.08
NewTom VGi evo	110	3	4.32	5 × 5	0.1
Veraview X800	90	5	17.86	4 × 4	0.08

Abbreviations: CBCT, cone‐beam computed tomography; cm, centimeter; FOV, field of view; kVp, kilovoltage peak; mA, milliamperes; mm, millimeter; s, seconds.

Prior to each CBCT scan, a crown made of each material tested was individually placed on the right mandibular second premolar. Additionally, two extra CBCT acquisitions within each protocol were conducted to minimize the impact of electric current variations. As such, there were a total of 54 volumes (two crown materials × three CBCT systems × three acquisition protocols × three repetitions).

For the reference standard, images of all the crowns were acquired using a micro‐computed tomography system (SkyScan 1278; Bruker), with the following protocols: 50 kV, 796 uA, 60 ms, 50 µm, 1 mm aluminum filter, and 0.7 rotation step.

**Figure 3:**
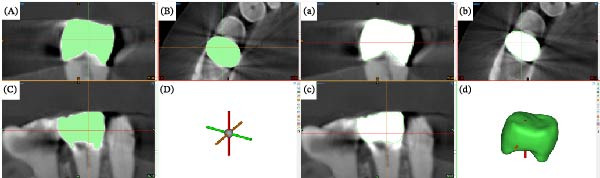
Segmentation of the zirconia crown with the medium field of view with standard resolution protocol from the 3D Accuitomo 170 system. (A–D) Mask after manual refinement in axial, coronal, and sagittal view. (a–d) Generation of the 3D object. (A, a) Coronal view. (B, b) Axial view. (C, c) Sagittal view. (d) 3D object.

The evaluators were calibrated through an introduction to the necessary software and its features, as well as explanation of the steps required for acquisition of the data used in this study. As such, all acquired images were imported into a medical imaging toolbox software program (Mimics Medical 25.0; Materialise) in Digital Imaging and Communications in Medicine (DICOM) format. The segmentation of the crowns was performed by one operator (S.M.M) using a semi‐automatic approach, in which masks were generated based on thresholding and then refined manually when necessary (Figure 3A–D). Because gray‐value intensity varied between acquisition protocols and CBCT systems, the operator visually inspected the initial thresholding output for each DICOM dataset to define the most suitable threshold. To ensure consistency and avoid subjective variability in the process, the operator followed the same decision‐making criteria for all samples, selecting the threshold that maximized crown capture while minimizing inclusion of adjacent structures. This standardized visual assessment strategy ensured reproducibility across the entire dataset. Afterward, the 3D objects (Figure 3a–d) were exported in STL format.

**Figure 4:**
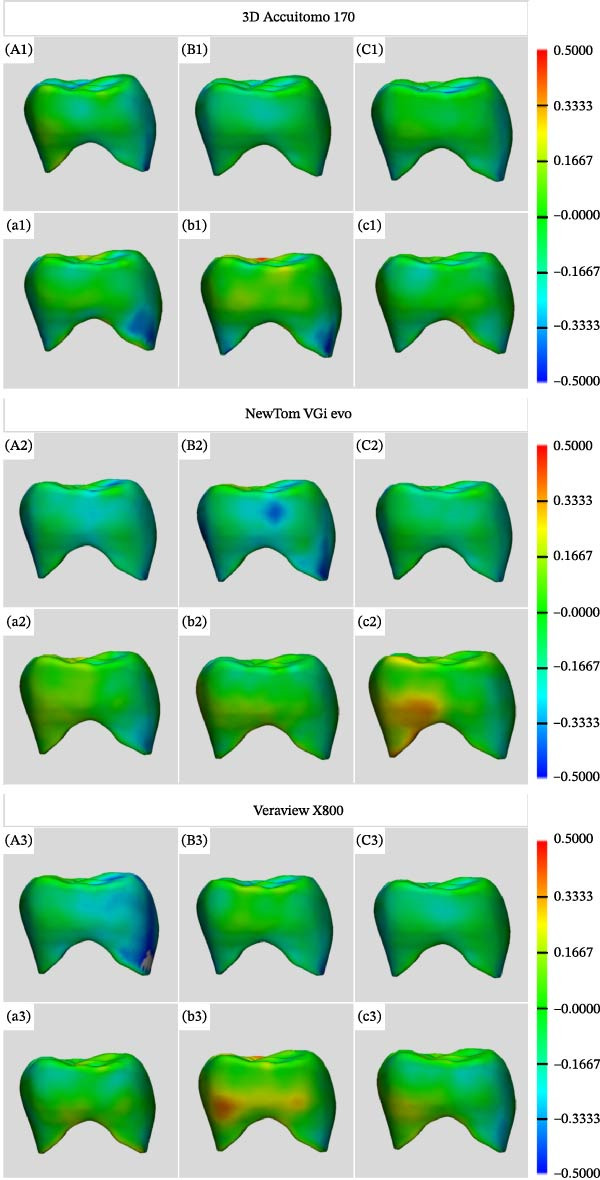
Color‐coded visualization of part comparison analysis in millimeters in a distal view of the crowns. Uppercase letters indicate the graphene‐reinforced material. Lowercase letters indicate the zirconia material. (A1–A3, a1–a3) Medium field of view (FOV) with standard resolution. (B1–B3, b1–b3) Small FOV with standard resolution. (C1–C3, c1–c3) Small FOV with high resolution. Green colors indicate little to no difference. Blue colors indicate study STL smaller than the reference SLT. Red colors indicate study STL larger than the reference STL.

For evaluation, the STL files of the segmented crowns were imported into a 3D printing, design, and remeshing software (3‐matic Medical 17.0; Materialise). The volume and surface area of each study STL and their respective reference STL were obtained, and the absolute differences between these measurements were calculated for further analysis. Using the same software, the N Points Registration semi‐automatic feature aligned each study STL with the reference STL. The Part comparison analysis with a color‐coded map visually represented the differences, and the distance maps (Euclidean distance) between the surfaces of each study STL and their respective reference STL were generated (Figure 4). The distance maps illustrate the root mean square error (RMSE) for each comparison, in which values closer to 0 indicate minimal displacement of points between the study and the reference STL files. Consequently, these variables quantitatively expressed the following outcomes for each CBCT system: volumetric alteration artifact (volume differences), surface area distortion (surface area differences), and general artifact expression (RMSE values).

To characterize overall artifact intensity, RMSE was adopted as the primary metric of general artifact expression, reflecting the degree of deviation between CBCT‐scanned crowns and the micro‐CT reference volumes. Higher RMSE values indicate greater volumetric distortion and more severe artifact expression. This allows for a single, comprehensive measure to compare the influence of different crown materials and imaging protocols on artifact formation.

The volumetric alteration artifact, surface area distortion, and general artifact expression were summarized using mean, median, standard deviation, and 95% confidence interval. A factorial two‐way repeated measures analysis of variance (ANOVA) was used to determine whether these outcomes were influenced by the factors under study: material, acquisition protocol, and their interactions. Additionally, a Tukey post hoc analysis was used for pairwise comparisons between the groups with a significance level set at 5%. The ANOVA and Tukey post hoc analyses were conducted with a statistical software program (Jamovi 2.3.28; Jamovi project).

Thirty days after the initial image evaluation, 30% of the sample was reevaluated across all material–machine–protocol combinations to analyze intra‐ and inter‐examiner reproducibility. A second operator (F.B.F.) participated in the inter‐examiner reproducibility analysis, following the same methodological steps previously described for segmentation and data evaluation. Intraclass correlation coefficient (ICC) was used to analyze both reproducibility assessments using SPSS 20 software (IBM Corporation).

Post hoc analysis demonstrated that this study achieved a statistical power of 0.93 (effect size = 0.3, *α* = 0.05; 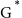
Power 3.1.9.7; Universitat Kiel, Germany).

## 3. Results

The ICC values demonstrated excellent reproducibility for intra‐ and inter‐examiner analysis (0.990 and 0.989, respectively) [43].

The mean, standard deviation, and ANOVA results are presented on Table 2 (volumetric alteration artifact), Table 3 (surface area distortion), and Table 4 (general artifact expression). The zirconia crown presented significantly more volumetric alteration artifact, surface area distortion, and general artifact expression than the graphene‐reinforced crown for the 3D Accuitomo 170 (*p* = 0.002, *p* = 0.003, and *p*  < 0.001, respectively) and NewTom VGi evo systems (*p* < 0.001, *p* = 0.007, and *p*  < 0.001, respectively). The acquisition protocols tested did not impact the volumetric alteration artifact (*p*  > 0.05; Table 2). However, for the Veraview X800 system, the zirconia crown scanned with both the small SR and small HR protocols presented more surface area distortion than the graphene‐reinforced crown scanned with the small SR protocol (*p* = 0.01 and *p* = 0.028, respectively; Table 3). For the same system and both materials, the medium SR protocol presented greater general artifact expression than the small SR protocol (*p* = 0.004; Table 4).

**Table 2:** Mean and standard deviation for the volumetric alteration artifact (mm^3^) according to material, acquisition protocol, and CBCT system.

Material	Protocol	CBCT system		
		3D Accuitomo 170	NewTom VGi evo	Veraview X800
		Mean (SD)	Mean (SD)	Mean (SD)
Zirconia^A^	Medium SR	65.1 (3.94)	65.5 (4.1)	66.4 (7.16)
	Small SR	57.1 (3.38)	62.7 (1.14)	51.5 (5.18)
	Small HR	55.1 (2.69)	51.4 (2.64)	52.3 (3.88)
Graphene^a^	Medium SR	20.8 (6.21)	4.39 (3.11)	1.30 (3.38)
	Small SR	8.86 (2.57)	−4.86 (7.28)	−11.1 (4.47)
	Small HR	11.1 (6.41)	−5.2 (1.64)	−6.37 (6.05)

*Note*: (A, a), Differences between superscript uppercase and lowercase letters indicate statistically significant differences for the 3D Accuitomo 170, NewTom VGi evo, and Veraview X800 systems (*p* = 0.002, *p*  < 0.001, and *p*  < 0.001, respectively).

Abbreviations: CBCT, cone‐beam computed tomography; SD, standard deviation.

**Table 3:** Mean and standard deviation for the surface area distortion (mm) according to material, acquisition protocol, and CBCT system.

Material	Protocol	CBCT system		
		3D Accuitomo 170	NewTom VGi evo	Veraview X800
		Mean (SD)	Mean (SD)	Mean (SD)
Zirconia	Medium SR	−12.2 (2.81)^A^	−7.18 (2.25)^A^	−9.84 (3.08)^BC^
	Small SR	−15.5 (0.86)^A^	−9.84 (1.73)^A^	−15.28 (1.14)^B^
	Small HR	−16.4 (1.66)^A^	−12.8 (3.35)^A^	−15.7 (0.28)^B^
Graphene	Medium SR	4.31 (2.64)^a^	−4.92 (1.57)^a^	−8.86 (2.47)^BC^
	Small SR	4.83 (2.12)^a^	−6.92 (4.2)^a^	−6.69 (1.23)^C^
	Small HR	8.72 (4.99)^a^	−8.09 (1.87)^a^	−3.88 (3.75)^BC^

*Note:* (A, a) Differences between superscript uppercase and lowercase letters indicate statistically significant differences between the materials for the 3D Accuitomo 170 and NewTom VGi evo systems (*p* = 0.003 and *p* = 0.007, respectively). (B, C) Different superscript uppercase letters indicate statistically significant differences between the combination of graphene crowns scanned with small SR protocol and zirconia crowns scanned with small SR and small HR protocols for the Veraview X800 system (*p* = 0.01 and *p* = 0.028, respectively).

Abbreviations: CBCT, cone‐beam computed tomography; SD, standard deviation.

**Table 4:** Mean and standard deviation for the general artifact expression (mm) according to material, acquisition protocol, and CBCT system.

Material	Protocol	CBCT system		
		3D Accuitomo 170	NewTom VGi evo	Veraview X800
		Mean (SD)	Mean (SD)	Mean (SD)
Zirconia^A^	Medium SR	0.3 (0.02)	0.30 (0.01)	0.33 (0.01)^B^
	Small SR	0.28 (0.01)	0.30 (0.01)	0.26 (0.02)^C^
	Small HR	0.28 (0.02)	0.26 (0.03)	0.28 (0.02)^BC^
Graphene^a^	Medium SR	0.17 (0.02)	0.12 (0.02)	0.15 (0.01)^B^
	Small SR	0.16 (0.03)	0.12 (0.02)	0.13 (0.01)^C^
	Small HR	0.13 (0.03)	−5.2 (1.64)	0.11 (0.01)^BC^

*Note:* (A, a) Differences between superscript uppercase and lowercase letters indicate statistically significant differences for every CBCT system (*p* < 0.001). (B, C) Different superscript uppercase letters indicate statistically significant differences between the medium SR and small SR protocols for the Veraview X800 system (*p* = 0.004).

Abbreviations: CBCT, cone‐beam computed tomography; SD, standard deviation.

## 4. Discussion

In CBCT imaging, the material composition of dental restorations plays a critical role in image quality and measurement accuracy. High‐density materials, such as zirconia, can produce pronounced volumetric and surface distortions, which may obscure peri‐crown and peri‐implant tissues and lead to misinterpretation of bone defects, secondary caries, or implant interfaces. In contrast, lower‐density materials like graphene generate substantially fewer artifacts, preserving tissue visibility and enabling more reliable volumetric assessments. These observations underscore the importance of considering crown material when interpreting CBCT scans and highlight the need to optimize imaging protocols, particularly for the evaluation of high‐density crowns.

The data collected in this study partially supported our hypotheses, since the zirconia crown exhibited more volumetric alteration artifact, surface area distortion, and general artifact expression than the graphene‐reinforced crowns. However, the influence of FOV and voxel size on artifact expression varied among the CBCT systems.

The evaluation of crowns in CBCT images is essential for the detection of secondary caries [44, 45], and marginal discrepancies that may not be identified in conventional imaging. Although some high‐density materials, such as zirconia, can generate beam hardening artifacts that compromise image quality, CBCT remains a valuable tool for 3D assessment of surrounding anatomical structures, aiding in diagnosis, treatment planning, and long‐term clinical monitoring.

Besides, studies demonstrate that artifacts can impact different tasks during clinical practice [46], including segmentation of objects [47]. Segmentation is an important step in areas, such as oral and maxillofacial surgery and prosthodontics, in which the development of a virtual patient directly correlates with treatment planning. As such, it is necessary to evaluate different artifacts that may interfere with the mentioned tasks.

Following general principles of transparent and reproducible experimental studies, the methodology in this study was reported through the steps of phantom preparation, image acquisition, dental crown segmentation, registration of study and reference crown, data collection, and statistical analysis. The phantom preparation and image acquisitions were performed by specialists according to what is established in the literature for prosthodontics and dentomaxillofacial radiology [48]. Al‐Rimawi et al. [49]’s study was used as a base for the segmentation and registration steps, but with the use of different software for segmentation.

In this study, the experimental groups were paired due to the use of the same crown throughout the CBCT scannings, which increases the robustness of the statistical analyses and reduces the required sample size. Considering the variables remain consistent throughout the scanned repetitions, the differences are assumed to derive from potential fluctuations that interfere with X‐ray production, such as electrical fluctuations, and from the segmentation and registration steps. Since the ICC values demonstrated excellent reproducibility for intra‐ and inter‐examiner comparisons, repeated measures ANOVA was used to evaluate at least three repetitions of the same object, and a post hoc analysis demonstrated statistical power of 0.93, three repetitions were deemed sufficient for this study.

RMSE was employed as the primary indicator of general artifact expression, quantifying the deviation between CBCT‐reconstructed volumes and the micro‐CT reference. Higher RMSE values denote increased gray‐value inconsistency and volumetric distortion and, therefore, offer a single comparative metric to evaluate how different crown materials and acquisition protocols influence artifact formation. Previous work has shown that excessive RMSE‐derived deviation can interfere with diagnostic accuracy, although the threshold considered acceptable varies depending on the intended clinical application [50].

While volumetric alteration, surface distortion, and RMSE describe distinct dimensions of artifact behavior, interpreting them collectively provides a more complete picture of how reconstruction errors affect image fidelity. In this integrated context, higher artifact expression reflects loss of geometric and density integrity [4, 9–11], which may compromise visualization of fine structures adjacent to high‐density crowns. Clinically, such distortion has the potential to obscure early secondary caries or reduce perceptibility of crestal bone changes in peri‐implant evaluation, delaying diagnosis and impairing treatment planning.

Previous studies suggest that the choice of material [7, 16, 17], acquisition protocol [8, 16, 39], and CBCT system [15] can interfere with the volumetric alteration artifact expression. Regarding the material, Table 2 descriptively shows an increased volume for both zirconia and graphene‐reinforced crowns compared to the reference standard, with zirconia showing higher values. ANOVA confirmed a significant difference between the materials, indicating that zirconia crowns exhibited more volumetric alteration artifact. Similarly, Table 4 illustrates that the zirconia crown had increased general artifact expression than the graphene‐reinforced crown. While no previous studies have directly compared volumetric alteration artifacts between zirconia and graphene‐reinforced crowns, the results of this study corroborate previous findings indicating that objects made with zirconia produce more artifact expression [7, 16, 26], while those made from graphene do not exhibit similar artifacts [35]. Additionally, a previous study comparing fiber‐reinforced composite with titanium orbit floor implants also showed less artifact generation from the former—a low‐density material [51].

The surface area distortion was significantly higher for zirconia than the graphene‐reinforced crowns (*p*  < 0.05; Table 3). Additionally, a descriptive analysis revealed that the absolute values of surface area distortion for zirconia were negative and lower than those obtained for the graphene‐reinforced crown. This finding, coupled with the results from Table 2, indicates that while zirconia restorations resulted in increased crown volume, these consistently exhibited smaller surface areas. This discrepancy may suggest a loss of surface details in the zirconia crowns compared to both the reference standard and the graphene‐reinforced crowns. In fact, during the study, a subjective observation of the CBCT images indicated that the graphene‐reinforced crowns were more similar with the reference standard than the zirconia counterpart, which hardly showed anatomical landmarks on the occlusal surface. Although no published studies have correlated surface area with surface detail when evaluating artifact expression in CBCT scans, we believe it is possible that this loss negatively impacts diagnostic tasks in which the morphology of the object is an important factor for evaluation, such as segmentation for virtual planning in oral and maxillofacial surgery and prosthodontics.

While commercially available zirconia materials may exhibit variations in composition, their primary constituent atoms are the ones used to identify the material: zirconium. The atomic number of zirconium corresponds to 40. In contrast, graphene primarily consists of carbon, which has an atomic number of 6. Additionally, the physical density of zirconia is approximately 6.1 g/cm^3^ [7], whereas graphene has a lower density of 2.27 g/cm^3^ [52]. These properties elucidate the distinctions between zirconia and graphene in CBCT artifacts expression, as demonstrated in this study.

Regarding the acquisition protocols, the present study’s results showed no influence of this parameter on the volumetric alteration artifact for any material or CBCT system studied. These consistent results suggest that while FOV and voxel size varied slightly across systems and protocols, they did not significantly influence the measured volumetric alteration artifact in this study.

To the best of our knowledge, only one previous study has assessed volumetric distortion artifacts in implants by measuring linear distortions at different levels of a titanium implant (i.e., a high‐density material) [15]. No previous study has investigated this phenomenon in dental crowns, highlighting the clinical relevance of the present study. That study reported results similar to ours for the 3D Accuitomo 170 system with the same acquisition parameters. However, it found greater volumetric artifact alteration with the NewTom VGi evo system using the small FOV SR protocol and with the Veraview X800 system using the medium and small FOV SR protocols. Despite similar acquisition protocols being employed, it is plausible that the volumetric alteration artifact of titanium behaves differently to that of zirconia and graphene‐reinforced material. This could explain the slight discrepancies observed between these studies.

Similarly to the findings of the present study, a previous one [16] showed no influence of FOV on the volumetric alteration artifact of zirconium cylinders at a constant 0.2‐mm voxel size for the OP300 and ProMax 3D systems. However, with voxel sizes similar with the ones used in the present study (0.085, 0.125, and 0.2 mm) and a small FOV (5 × 5 cm) for the OP300 system, these authors showed increased volumetric alteration artifact of the zirconium cylinder as voxel sizes increased. The morphology of the scanned object or system specificities, such as bit depth, might have influenced the differences observed in this study.

For instance, the OP300 system has a bit depth of 13, whereas the systems used in this study have a bit depth of 14 or higher. An increased bit depth means higher contrast resolution, which leads to a broader range of gray values on the CBCT images [53]. This enhancement in contrast resolution is particularly advantageous for imaging high‐density materials, potentially affecting the appearance of volumetric alteration artifacts [7]. Image quality, however, is affected by a variety of characteristics, including technical aspects of the CBCT system—such as sensor quality and signal‐to‐noise ratio [1]. Therefore, bit depth can lead to improved image quality provided other technical characteristics of the systems can harness the different gray values.

For the surface area distortion and general artifact expression, however, the acquisition parameters had an influence on the Veraview X800 system. The hybrid nature of this system in comparison with a dedicated CBCT system from the same manufacturer (3D Accuitomo 170) may explain the differences observed, as the dedicated system is highly stable and can more easily compensate for artifacts.

As such, regarding surface area distortion on the Veraview X800 system, the zirconia crown scanned using the small FOV SR protocol presented more distortion than the graphene‐reinforced crown scanned under both the small FOV SR and small FOV HR protocols (Table 3). Meanwhile, the medium FOV SR presented more general artifact expression than the small FOV SR (Table 4), which aligns with findings from previous studies [39, 40]. Significant differences in artifact expression for CBCT‐specific variations in acquisition parameters may be related to disparities in image acquisition and reconstruction [15, 54].

Micro‐computed tomography is widely regarded as the gold standard for morphological analysis of dental tissues, as it provides micrometric resolution, high dimensional accuracy, and minimal expression of imaging artifacts [8]. These features make it a reliable reference for validating the diagnostic performance and measurement accuracy of CBCT scans. Due to the repetitiveness of CBCT acquisitions, this study had the limitation of not being conducted in a real clinical scenario. Consequently, there were no motion artifacts, which can significantly degrade the resolution of CBCT scans [1]. especially when combined with beam hardening artifacts. However, this study’s phantom consisted of a dry human skull, mandible, teeth, and soft tissue‐simulating material to simulate a clinical scenario as much as possible [41].

Although the impact of zirconia artifacts on CBCT imaging is well established, this study is the first to evaluate the material in the actual geometry of a crown, which adds clinical relevance compared to previous assessments with simplified shapes. Despite zirconia‐related artifacts being problematic and contributing to additional radiation exposure, CBCT exams may still be required in scenarios such as detecting alterations adjacent to prosthetic crowns or assessing peri‐implant and other osseous defects [55].

Furthermore, graphene‐reinforced crowns are not commonly used in daily clinical practice. However, studies explore the potential of graphene in prosthodontics, especially its combination with PMMA, a resin widely used in prosthetic treatments [29, 30, 32–34, 56, 57]. There are also signs that PMMA shows improved fracture resistance when reinforced with graphene [31, 58]. Therefore, despite the absence of graphene‐reinforced prosthetic crowns in the clinical scenario, it was necessary to evaluate its radiologic features independently.

In this study, the MAR algorithm was intentionally not activated to avoid algorithm‐related variability and enable direct assessment of inherent streaking and beam‐hardening patterns. It is, however, important to recognize that MAR tools could alter the visualization of high‐density restorative materials. When activated, MAR algorithms may attenuate radiopaque streaks, homogenize gray‐value distribution, and potentially improve interpretability in clinical scenarios involving zirconia or other dense ceramics [14, 59–61]. However, image smoothing and edge blurring have also been reported as undesirable consequences, which may obscure fine structural details or reduce diagnostic sharpness [62]. In fact, previous studies demonstrated that the effectiveness of MAR algorithms can greatly vary due to the many factors that influence it [63, 64].

Although zirconia is already well established as an emerging restorative material, graphene has recently gained attention for its favorable physical and radiographic behavior. Evidences from our study suggest that lower‐density materials, such as graphene‐reinforced biopolymers, produce less pronounced volumetric alteration artifacts, surface distortion, and general artifact expression in CBCT scans, even when evaluated across different systems and acquisition protocols. These findings support the continued investigation of graphene and other similar‐density materials with the aim of future clinical adoption.

However, current knowledge regarding graphene’s long‐term mechanical durability, stability of esthetic properties (e.g., color and gloss), and cost‐effectiveness in a clinical scenario remains limited due to lack of related studies. Longitudinal and comparative studies are, therefore, needed to determine its viability in clinical settings. Additionally, further analysis using different CT systems, evaluating exomass configuration, and object positioning within the FOV may broaden understanding of artifact behavior. Finally, studies focusing on specific diagnostic tasks and on how quantitative differences in volumetric alteration, surface distortion, and RMSE‐based artifact expression affects clinical interpretation would help clarify the practical implications of both materials.

## 5. Conclusion

Based on the findings of this ex vivo study, the following conclusions were drawn:

1. Zirconia exhibits greater volumetric alteration artifacts, surface area distortion, and general artifact expression compared to the graphene‐reinforced material, while presenting a reduced surface area consistent with loss of surface detail in the crown morphology. Thus, reinforcing the potential benefits of lower‐density restorative materials, such as graphene‐enhanced materials for dental applications, after a thorough clinical validation.
2. Specific CBCT systems and parameter protocols might affect artifact expression and surface area distortion independently of using a high‐ or low‐density material.

## Author Contributions

**Sâmia Mouzinho-Machado:** conceptualization, data curation, formal analysis, investigation, methodology, writing – original draft, writing – review and editing. **Fernanda Bulhões Fagundes:** data curation, investigation, writing – review and editing. **Rocharles Cavalcante Fontenele:** conceptualization, investigation, methodology, writing – original draft, writing – review and editing. **Bahaaeldeen M. Elgarba:** methodology, resources, writing – original draft. **Reinhilde Jacobs:** conceptualization, data curation, methodology, project administration, resources, supervision, writing – review and editing. **Sergio Lins de-Azevedo-Vaz:** conceptualization, formal analysis, methodology, project administration, supervision, writing – original draft, writing – review and editing.

## Acknowledgments

Graphenano Dental supplied the G‐CAM disk used for the graphene‐reinforced crown confection.

## Funding

This study was financed in part by the Coordenação de Aperfeiçoamento de Pessoal de Nível Superior–Brasil (CAPES), Finance Code 001.

## Conflicts of Interest

The authors declare no conflicts of interest.

## Data Availability

The data that support the findings of this study are available from the corresponding author upon reasonable request.
